# Polish Adaptation and Psychometric Validation of the PREM-C9 Questionnaire for Patients with Chronic Obstructive Pulmonary Disease

**DOI:** 10.3390/healthcare11202746

**Published:** 2023-10-16

**Authors:** Iwona Damps-Konstańska, Weronika Ciećko, Ewa Jassem, Tomasz Bandurski, Dominika Bosek, Marzena Olszewska-Karaban, Ewa Bandurska

**Affiliations:** 1 Division of Allergology, Medical University of Gdańsk, 80-210 Gdańsk, Poland; 2 Center for Competence Development, Integrated Care and e-Health, Medical University of Gdańsk, 80-210 Gdańsk, Poland; weronika.ciecko@gumed.edu.pl (W.C.); dominika.bosek@gumed.edu.pl (D.B.); ewa.bandurska@gumed.edu.pl (E.B.); 3 Division of Pulmonology, Medical University of Gdańsk, 80-210 Gdańsk, Poland; ewa.jassem@gumed.edu.pl; 4 Division of Radiology Informatics and Statistics, Medical University of Gdańsk, 80-210 Gdańsk, Poland; tomasz.bandurski@gumed.edu.pl; 5 Division of Rehabilitation Medicine, Medical University of Gdańsk, 80-210 Gdańsk, Poland; marzena.olszewska-karaban@gumed.edu.pl

**Keywords:** chronic obstructive pulmonary disease (COPD), patient-reported outcome measures, healthcare surveys, PREMC-9

## Abstract

**Introduction**: Chronic obstructive pulmonary disease (COPD) is a common, preventable, and treatable disease. The first PREM (patient-reported outcome measure)-type questionnaire that has been dedicated to assess the experience of care in COPD is the PREM-C9. **Aim**: The aim of this study was to create a Polish version of the PREM-C9 and determine its psychometric characteristics. **Methods**: The validation procedure involved forward and back translation. We included 42 patients with COPD. The psychometric properties were assessed using Cronbach’s alpha, Bartlett’s test, the Kaiser–Meyer–Olkin test, and Spearman’s correlation coefficient. The validity of the questionnaire was assessed using a principal component analysis for the extracted principal components. The validity of the factor analysis was demonstrated using Bartlett’s sphericity test and the Kaiser–Meyer–Olkin (KMO) test. A factor analysis was performed using the Oblimin and Varimax rotation. The reliability of the questionnaire was assessed using Cronbach’s alpha. **Results**: The Polish version of the analyzed questionnaire met all the validation criteria: face, translation, psychometric, functional, and reconstruction equivalence. Spearman’s correlation results between the Polish PREM-C9 and CAT were as follows: rho = 0.44, *p* = 0.003539; HADS-Anxiety: rho = 0.370864, *p* = 0.015612; and HADS-Depression: rho = 0.387405, *p* = 0.011253. **Conclusions**: The developed Polish PREM-C9 questionnaire is a reliable and valid tool that assesses Polish COPD patients’ experiences of their disease and the care they receive.

## 1. Introduction

Chronic obstructive pulmonary disease is a common, preventable, and treatable disease. About 384 million people worldwide are affected by COPD. According to the latest GOLD report, COPD is “a heterogeneous lung condition characterized by chronic respiratory symptoms (dyspnea, cough, expectoration, exacerbations) due to abnormalities of the airways (bronchitis, bronchiolitis) and/or alveoli (emphysema) that cause persistent, often progressive, airflow obstruction” [1]. The causes and risk factors of COPD have been recently described with the GETomic acronym, which stands for dynamic, cumulative, and repeated gene (G)–environment (E) interactions over a person’s lifetime (T) that damage their lungs or change their normal ageing processes [2]. The most important environmental risk factor is cigarette smoking, but this group also includes occupational exposure [3] and air pollution [4].

Commonly, assessments of the clinical effectiveness of interventions use well-defined endpoints. According to the guidelines of the Polish Agency for Health Technology Assessment, these are endpoints related to mortality, morbidity (related to the course/exacerbation of the disease), the health-related quality of life (HRQoL), unwanted events, and adverse effects (divided into severe and other) [5]. However, the same guideline underlines that endpoints relevant from the patient’s perspective should also be considered. These endpoints include patient-reported outcome measures (PROMs) and patient-reported experience measures (PREMs). The patient’s perspective is paramount. Principle 5 of the COPD Patients Charter refers to the patient’s right to appropriate specialist care whenever the patient needs it [6]. Improving patient care requires using appropriate tools to assess the care for patients with COPD.

According to the Food and Drug Administration (FDA), PROMs can be defined as “any reports of the status of a patient’s health condition that comes directly from the patient (without interpretation of the patient’s response by a clinician or anyone else)” [7]. Such information is collected through standardized questionnaires. These endpoints were originally developed and implemented for use in clinical trials as a way to include the patient’s voice in the evaluation of clinical interventions [8]. However, their use has gradually become more common, and today, they are considered an integral part of assessing clinical effectiveness and patient-centered care [9].

The second type of endpoints are PREMs. They allow for the patient’s perception of their experience with the received care to be determined. Typically, they are structured questionnaires, but unlike PROMs, they do not assess care outcomes, but determine how patients perceive their experience of using care. They can be used as indicators of the quality of care (determined from the patients’ perspective) at any level of healthcare system [10] and used by entitled agencies to assess medical technologies in the reimbursement process [11].

Like PROMs, PREMs also deliver information on the quality of care during the intervention, which allows for an immediate response and adjustments. Several publications have shown that considering patient experiences is a key factor in strengthening healthcare systems. These data may be used as a basis to identify quality, performance, or security issues [12,13].

The first PREM-type questionnaire that has been dedicated to assess the experience of care in COPD is the PREM-C9 [14]. It is a simple questionnaire validated for use in the population of patients with mild to severe COPD. However, this tool has not been validated under Polish conditions yet. Therefore, the study aims are as follows:

- To create a Polish adaptation of the PREM-C9.
- To determine the psychometric characteristics of the questionnaire used under Polish conditions.
- To provide knowledge regarding the characteristics and the role of the PREM-C9 in non-English-speaking countries, allowing for cross-cultural comparisons, determining further possibilities of using it, and generalizing the results obtained to other populations.

It is believed that the Polish validation of the PREM-C9 test will stimulate Polish research on the role of the patient’s perspective in the treatment of COPD.

Determining the usefulness of this questionnaire requires an examination of its validity and reliability. Thus, these parameters were assessed in a study on the Polish version of the PREM-C9. Questionnaire validity is understood as the level of agreement with which the assessed questionnaire measures what it was designed for. Reliability is defined as a questionnaire’s ability to reflect the true value of the characteristic that has been evaluated [15].

## 2. Materials and Methods

### 2.1. The Adaptation Procedure 

The PREM-C9 questionnaire was developed by Hodson et al. [14]. The procedure involved the use of both forward and back translation methods in accordance with the ISPOR guidelines [16] and Breslin [17].

1. Forward translation of the PREM-C9 into Polish by a professional bilingual translator with Polish as a native language.
2. Reviewing the translated version of the PREM-C9 by the team, including pneumonologists and the research team members. This stage did not provide any significant changes to the Polish version of the questionnaire.
3. Back translation made by a professional bilingual translator with English as a native language.
4. Comparison of the back-translated version with the original text. This stage did not provide any significant changes; only minor revisions were introduced.
5. Evaluation of the comprehensibility of the translated version in a group of 20 healthy volunteers who assessed whether each of the questions was understandable to them.
6. The first use of the PREM-C9 questionnaire in a group of 42 patients with COPD to validate the tool and test its psychometric features. The material was collected from patients by a trained interviewer, or patients answered by themselves by filling out a paper version of the questionnaire. The questionnaire was made available both in paper form and in a digital version. The use of the electronic version of the questionnaire was caused by the COVID-19 pandemic and resulted from the need to limit interpersonal contacts.

**Figure 1:**
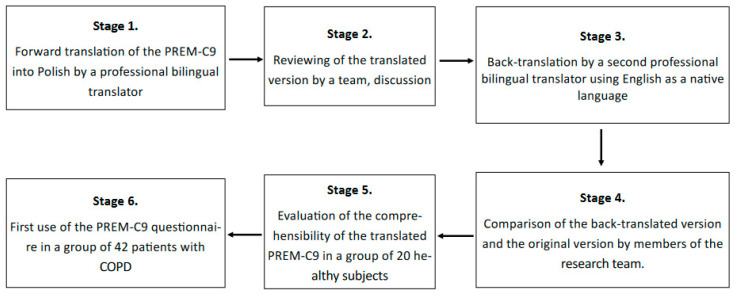
Stages of the adaptation procedure of the PREM-C9 questionnaire.

**Figure 2:**
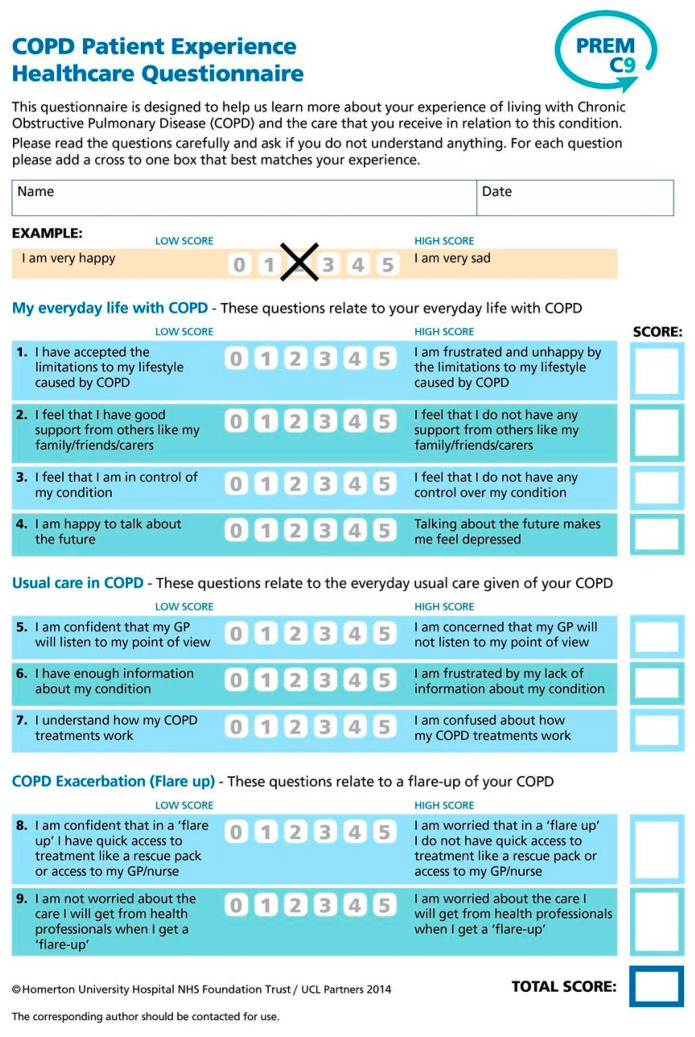
The English and Polish version of PREM-C9.

The result of conducting the adaptation procedure was the preparation of the Polish version of the PREM-C9 questionnaire, which was used for further testing of the adapted version. The adaptation procedure is presented in Figure 1, and the translated questionnaire is in Figure 2. 

### 2.2. Participants

The study was approved by the bioethics committee (NKBBN/211/2021) of the Medical University of Gdańsk, and consent was obtained from the first author of the validated questionnaire for its use. In addition, permission to use the other questionnaires in the study was obtained as well.

A total of 42 Polish-speaking participants were tested. The inclusion criteria were as follows:

- A patient diagnosed with COPD according to the GOLD criteria from 2022;
- A patient expressing informed consent to participate in the study;
- A patient not hospitalized at the time of the study due to COPD exacerbation or other reasons;
- A patient who was able to answer questions.

Most of the patients participating in the study (*n* = 31; 73.81%) confirmed that dyspnea makes them walk slower than their peers or makes them stop to catch a breath when they walk at their own pace. According to the mMRC (Modified British Medical Research Council) [18], this is equivalent to at least grade 2 dyspnoea. The basic characteristics of the study group with their socio-demographic characteristics are presented below (Table 1).

### 2.3. Measures

**The patient-reported experience measure in chronic obstructive pulmonary disease PREM-C9** (hereinafter also abbreviated as PC-9) is a simple, 9-item tool of the relational PREM type that is divided into three sections:

- My everyday life with COPD (4 questions);
- Usual care in COPD (3 questions);
- COPD exacerbations/flare-ups (2 questions).

The patient evaluates the statements using a graphical Likert scale with the two extreme labels marked as 0 (the lowest score), which describes the best situation, and 5 (the highest score), dedicated to the worst situation. In the Polish adaptation and validation of the questionnaire, we used the same questionnaires as in the original validation process (CAT^TM^ and HADS), with one additional general PROM—EuroQol EQ-5D 5-level version (EQ-5D-5L). They are briefly described below. 

**The COPD assessment test (CAT^TM^)** [19] (Polish version) is a disease-specific PROM-type questionnaire dedicated to people with COPD that assesses how the disease impacts a person’s life and whether it changes over time. It consists of 8 items, and the answering scale is from 0 to 5, where 5 is the worst rating. The CAT considers the presence and severity of a cough, phlegm, a sense of tightness in the chest, breathlessness, limitations in performing everyday activities, self-confidence when leaving home, the quality of sleep, and the life energy level. A score of 5 points means the upper limit of normal in healthy non-smokers, <10 points means that COPD has a minor influence on the patient’s life, 10–20 points means there is an average impact, >20 means there is a severe impact, and >30 means there is a very severe impact—the disease makes it impossible to perform any everyday activities, and the patient never has good days. It is worth noting that the construction and graphic layout of PREM-C9 and CAT^TM^ are very similar. 

**The hospital anxiety and depression scale modified version (HADS-m)** [20] (Polish version) is a PROM-type questionnaire measuring anxiety and depression in patients who are somatically ill. It consists of three subscales—depression (HADS-D), anxiety (HADS-A), and irritability (two questions from the last subscale were not used in the original version, and subsequently, they were not used in this study either). The whole questionnaire consists of 16 questions, which are evaluated on a 0–3 scale. The obtained results can be interpreted as follows: ≤7 = normal, 8 to 10 = borderline abnormal (borderline case), and 11 or higher = abnormal (case). Since, in this study, only two subscales (A and D) were used, the questionnaire will hereafter be referred to as HADS (as the modification was the addition of the third unused subscale).

**The European quality of life scale 5D 5-level version (EQ-5D-5L)** [21] (Polish version) is a general PROM-type questionnaire consisting of 5 questions (considering: mobility, self-care, usual activities, pain and discomfort, and anxiety and depression) that can be evaluated using 5 levels: no problems (1 point), slight problems, moderate problems, severe problems, or extreme problems (5 points), and a visual analogue scale (EQ-VAS) to describe the general health status on the day of attending the study. The EQ-5D-5L is available in more than 150 languages and is one of the most frequently used generic questionnaires assessing the health-related quality of life (HRQoL) [22].

### 2.4. Statistical Methods and Calculations

All calculations were performed using the Microsoft Excel spreadsheet questionnaire, the StatSoft Inc. 14.0 statistical package Statistica, and the SPSS program, version 21. In the statistical description of the quantitative data, classical measures of position such as the arithmetic mean, median, and standard deviation were used as measures of the variability. To evaluate the psychometric properties of the PREM-C9 questionnaire, Cronbach’s alpha, Bartlett’s test of sphericity, the Kaiser–Meyer–Olkin coefficient, and Spearman’s correlation coefficient were used. In all statistical tests, *p* < 0.05 was taken as the level of statistical significance for differences.

The validity of the questionnaire was assessed using a PCA (principal component analysis) for the extracted principal components. The validity of the factor analysis was demonstrated using Bartlett’s sphericity test and the Kaiser–Meyer–Olkin (KMO) coefficient, with *p* < 0.05 and KMO > 0.6 as the cut-off levels, respectively. A factor analysis was performed using the Oblimin and Varimax rotation method, assuming 0.4 as the threshold value. The reliability of the questionnaire was assessed using Cronbach’s alpha index, assuming 0.70 as the cut-off value.

## 3. Results

The Polish language version of the questionnaire was named identically to the original version—PREM-C9. Like the original version, the Polish questionnaire consists of nine questions divided into three sections. Developing a Polish version was proceeded in accordance with the principle of facade equivalence (test graphics, instructions).

This questionnaire and the Polish versions of the CAT, HADS, and EQ-5D-5L questionnaires were given to the patients participating in the study for completion. The results obtained in the study group using the individual tests are presented in Table 2.

In the overall assessment of the current health status using the EQ-VAS, a mean score of 60.56% of the ideal value was obtained (Me = 60; SD = 22.32). The mean scores obtained for the whole group on the CAT scale should be interpreted as the average impact of COPD on patients’ lives, which was higher than in the well-controlled patients (CAT > 10 points). The results of the HADS scale indicated that, overall, patients did not seem to have depression or anxiety (mean score for the whole group < 8 points). However, a score greater than or equal to 8 was obtained for four patients on the HADS-D scale and six patients on the HADS-A scale, which should be interpreted as borderline abnormal or even abnormal values.

### 3.1. Assessment of Psychometric Characteristics 

#### Validity of the PREM-C9 Questionnaire

In order to examine the theoretical validity of the test, each part of the PREM-C9 was subjected to a factor analysis. The validity of the factor analysis was confirmed using Bartlett’s sphericity test, with a significant result (*p* = 0.000), which demonstrated the presence of a correlation between the components. In addition, the Kaiser–Meyer–Olkin (KMO) test results of >0.5 proved that there were relationships between the components and that they were selected appropriately, so the factor analysis was justified (Table 3).

The PCA (principal component analysis) factor extraction method using the Kaiser normalization criterion, with both Varimax and Oblimin rotation, identified three components (the same as in the English language version). In all the questions, a correlation value of at least 0.6 was obtained between the individual questions and the questionnaire as a whole, which means that each question in the Polish language version of the PREM-C9 is valid (contributes relevant content) and should not be removed. The lowest correlation value was obtained for question 4 (I am happy to talk about the future), but this was not below the accepted criterion and was noticeably higher when Oblimin was used as the rotation method. 

The next stage of the validity analysis was the convergent relevance analysis, for which Spearman’s correlation was used between the PREM-C9 questionnaire assessed and the other questionnaires, i.e., HADS, CAT, EQ-5D-5L, and EQ-VAS (Table 4). In each case, a statistically significant (*p* < 0.05) low or moderate correlation (a correlation of at least pronounced strength) was obtained.

### 3.2. The Reliability of PREM-C9 Questionnaire

The assessment of the reliability of the Polish version of the PREM-C9 questionnaire showed that Cronbach’s alpha index was 0.743, which confirms that the assessed questionnaire is reliable (value > 0.70). 

In order to analyze the correlation of individual items with the remaining considered as “a total”, the reliability assessment was repeated, but after removing individual questions from the questionnaire. It was shown that the elimination of individual statements did not significantly affect Cronbach’s alpha coefficient value for the rest of the questionnaire. The values obtained ranged from 0.690 to 0.767. The greatest reduction in the index was found when question 6 (I have enough information about my condition) was removed, which should be understood as the weakest correlation of this question with the others, but the value was still close to 0.70. The constructed correlation matrix of each question (Table 5) also showed that question 6, the removal of which resulted in the lowest value of Cronbach’s alpha, did not correlate significantly with the other questions. Question 2 was also the question that did not correlate with the others, but after removing it, Cronbach’s alpha only decreased by 0.032. However, as the aim of the study was not to modify the original tool, questions 6 and 2 were left in the Polish version, as in the original structure of the PREM-C9.

In addition, a reliability analysis using Klein’s criterion that individual variables should correlate with the total score for the whole scale at a level of at least 0.4 confirmed the reliability of the whole questionnaire, with all nine questions retained (Table 6). A comparison of Spearman’s rank order correlation results between PREM-C9, CAT, and HADS with the results of the original version (Hodson 2019) and the first use in the original language version (Jones 2020) is presented in Table 7.

## 4. Discussion

The Polish version of the PREM-C9 questionnaire met all the validation criteria, i.e., the face equivalence (test graphics, instructions), translation equivalence (question content), psychometric equivalence (similar correlation with questionnaires also used in psychometric analyses of the English language version of PREM-C9), functional equivalence (suitability for the same purposes), and reconstruction equivalence (checking reliability and validity). The validation of the PREM-C9 into Polish will allow the use of the questionnaire in the assessment of care for patients with COPD in Poland. The use of PREM indicators is still not a common practice, which previous studies have proven [24] and which limits the possibility of comparing the results obtained in terms of the accuracy and reliability of the Polish version of the PREM-C9 questionnaire with other works apart from the original assessment in the English language version. There are known studies using the PREM-C9 questionnaire in its original version. For example, a study by Jones et al. [23] measured patients’ experiences of living with COPD and the medical care they received, and the results were compared with other scales—some of which were also used in the validation study of the Polish language version (CAT and HADS). The correlation rho of the PREM-C9 (for all domains combined) with the CAT questionnaire score was 0.27 (thus, less than in the present study), but it was a statistically significant relationship (*p* = 0.03). However, only a result measuring towards significance was obtained for the correlation with the HADS-D subscale (*p* = 0.09). The correlation level obtained in the present study between the PREM-C9 test and the CAT questionnaire was 0.44, which is similar to the values obtained in the original validation of the tool (0.42). Similar results were also obtained for the HADS-A, and slightly lower results were obtained for the HADS-D [14]. In addition to the English language version, the PREM-C9 was translated into Catalan and Spanish [25]. Ten patients participated in this study; however, this study did not determine the questionnaire’s psychometric values, as the authors only translated it. Chaplin et al. defined a meaningful change in the PREM-9 following pulmonary rehabilitation. The change in the PREM-9 for responders (defined as a HADS anxiety MID ≥ −1.5) was −5.26 (SD: 8.33). A sensitivity and specificity analysis using an ROC with a HADS anxiety anchor yielded a change of −7.5 units. The minimum important difference for the PREM-9 was calculated between −3.67 and −7.5 units [26].

The PCA analysis using the Kaiser normalization criterion with Varimax and Oblimin rotation obtained the lowest correlation value for question 4 (I am happy to talk about the future). It should be acknowledged that the nature of the question itself may have influenced the result, as it is of a general type, going beyond the context of the disease itself; hence, it can be assumed that the results obtained for this question differed slightly from the others. 

An assessment of the reliability of the Polish version of the PREM-C9 questionnaire confirmed that it is a reliable tool and, therefore, correctly (accurately) reflects the actual condition. Cronbach’s alpha index was 0.743. According to various sources, even 0.6 can be considered a satisfactory level, although more often, it is 0.7 [27,28]. Slightly higher values were obtained in the validation of PREM-type tools dedicated to other conditions, e.g., rheumatic conditions [29], oncological conditions [30], and hypertension [31]. However, these were not studies conducted in a Polish setting; such studies, apart from this one, have not yet been produced. In addition, a literature review performed in Germany in 2022 showed that none of the PREM-type questionnaires analyzed were fully evaluated under German conditions [32].

## 5. Implications and Limitations of the Study

The benefits of using the patient perspective in shaping effective healthcare are well accepted. For example, the addition of these data in the care of patients with metastatic cancer to those collected in a standard way resulted in an increased survival compared to traditionally managed care [33], and in a group of patients with arthritis, it improved the self-perceived health [34]. Using PROM- and PREM-type data also reduces the utilization of healthcare system resources by improving symptom control, increasing patient satisfaction, and ultimately improving the HRQoL [35].

Much attention has been paid over the years to the reliability of questionnaires of the PROM and PREM type while at the same time pointing to the need to develop new validated research tools [36]. This study resulted in the development, in accordance with current principles, of a PREM-type questionnaire, the psychometric properties of which were meticulously examined and confirmed. This study may inspire and encourage other researchers to develop and use this type of questionnaire in patient care. What may be of note in the study is the small study group (42 patients). However, it should be clear that there are no specific guidelines on the minimum number of respondents for this type of study [37]. In the publication by Tsang et al. presenting guidelines for developing, translating, and validating questionnaires, guidance can be found suggesting that these processes should utilize between 30 and 50 people, and similar guidance can be found in a broader study by Aithal [38].

## 6. Conclusions

The developed Polish version of the PREM-C9 questionnaire is a reliable and valid tool that assesses Polish patients’ experiences of their disease (COPD) and the care they receive. 

The questionnaire can be used to conduct follow-up among Polish patients and for comparative studies in non-English-speaking countries, allowing for cross-cultural comparisons and the determination of further possibilities of using PREM-C9 and generalizing the results to other populations.

## Figures and Tables

**Table 1:** Characteristics of the clinical validation cohort: demographic data.

Parameter	Sub-Parameter	Number	Percent (%)
Gender	Female	21	50.00
	Male	21	50.00
Place of living	City	39	92.86
	Village	3	7.14
Education level	Primary	1	2.38
	Vocational	15	35.71
	Secondary	23	54.76
	Higher	3	7.14
Self-assessment of material status	Very good	7	16.57
	Average	25	59.52
	Poor	10	23.81
	Very poor	0	0.00
Living alone	Yes	11	26.19
	No	31	73.81

**Table 2:** Results according to separate questionnaires.

Questionnaire	The Average of the Points Obtained	% of Max Points (Showing The Worst State)	Median	SD
PREM-C9	9.33	20.73	6.5	6.98
CAT	17.02	42.63	16	8.90
EQ-5D-5L	10.07	40.28	9	4.27
HADS A (Anxiety)	4.09	19.48	3	4.05
HADS D (Depression)	3.29	15.67	2	3.27

**Table 3:** The results of Bartlett’s and KMO tests.

Kaiser–Meyer–Olkin Test and Bartlett’s Test		
KMO measure of sampling adequacy		0.675
Bartlett’s sphericity test	Approximated chi-squared value	108.382
	Df	36
	Significance	0.000

**Table 4:** Spearman’s correlation between PREM-C9 and EQ-5D-5L, EQ-VAS, HADS, and CAT.

Pair of Variables (Questionnaires)	Spearman’s Rank Order Correlation		
	N	Rho	*p*
CAT and PC-9	42	0.440	0.003
EQ-5D-5L and PC-9	42	0.387	0.011
EQ VAS and PC-9	41	−0.346	0.027
HADS-A and PC-9	42	0.371	0.015
HADS-D and PC-9	42	0.387	0.011

**Table 5:** Correlations between questions in PREM-C9 (upper row—correlation coefficient, lower row—*p*-value, Bold indicate significant correlations with *p* < 0.05).

	P-C9 1	P-C9 2	P-C9 3	P-C9 4	P-C9 5	P-C9 6	P-C9 7	P-C9 8	P-C9 9
P-C9 1		**0.51**	**0.48**	0.28	−0.07	**0.45**	**0.33**	0.06	0.25
		***p* = 0.001**	***p* = 0.001**	*p* = 0.072	*p* = 0.672	***p* = 0.003**	***p* = 0.034**	*p* = 0.723	*p* = 0.104
P-C9 2	**0.51**		**0.38**	0.16	0.15	**0.48**	0.15	0.20	0.16
	***p* = 0.001**		***p* = 0.014**	***p* = 0.313**	*p* = 0.351	***p* = 0.001**	*p* = 0.345	*p* = 0.211	*p* = 0.313
P-C9 3	**0**	**0**		**1**	0	**0**	0	0	**0**
	***p* = 0.001**	***p* = 0.014**		***p* = 0.000**	*p* = 0.566	***p* = 0.009**	*p* = 0.184	*p* = 0.108	***p* = 0.014**
P-C9 4	0.28	0.16	**0.54**		−0.14	0.26	0.08	−0.05	0.09
	*p* = 0.072	*p* = 0.313	***p* = 0.000**		*p* = 0.365	*p* = 0.102	*p* = 0.607	*p* = 0.755	*p* = 0.557
P-C9 5	−0.07	0.15	−0.09	−0.14		0.17	0.08	**0.43**	0.20
	*p* = 0.672	*p* = 0.351	*p* = 0.566	*p* = 0.365		*p* = 0.285	*p* = 0.614	***p* = 0.005**	*p* = 0.204
P-C9 6	**0.45**	**0.48**	**0.40**	0.26	0.17		**0.53**	0.27	**0.31**
	***p* = 0.003**	***p* = 0.001**	***p* = 0.009**	*p* = 0.102	*p* = 0.285		***p* = 0.000**	***p* = 0.081**	***p* = 0.046**
P-C9 7	**0.33**	0.15	0.21	0.08	0.08	**0.53**		**0.41**	**0.51**
	***p* = 0.034**	*p* = 0.345	*p* = 0.184	*p* = 0.607	*p* = 0.614	***p* = 0.000**		***p* = 0.006**	***p* = 0.001**
P-C9 8	0.06	0.20	0.25	−0.05	**0.43**	0.27	**0.41**		**0.48**
	*p* = 0.723	*p* = 0.211	*p* = 0.108	*p* = 0.755	***p* = 0.005**	*p* = 0.081	***p* = 0.006**		***p* = 0.001**
P-C9 9	0.25	0.16	**0.38**	0.09	0.20	**0.31**	**0.51**	**0.48**	
	*p* = 0.104	*p* = 0.313	***p* = 0.014**	*p* = 0.557	*p* = 0.204	***p* = 0.046**	***p* = 0.001**	***p* = 0.001**	

**Table 6:** Correlations of individual questions with the total score obtained for the entire test.

The Question Number in PREM-C9	Correlation with the Sum for P-C9	*p*
P-C9 1	0.6	0.000
P-C9 2	0.6	0.000
P-C9 3	0.7	0.000
P-C9 4	0.4	0.000
P-C9 5	0.4	0.000
P-C9 6	0.7	0.000
P-C9 7	0.6	0.000
P-C9 8	0.6	0.000
P-C9 9	0.6	0.000

**Table 7:** Summary of Spearman’s rank order correlation results for the Polish version of PREM-C9, CAT, and HADS with the results of the original version (Hodson 2019) and the first use in the original language version (Jones 2020).

Questionnaire	PREM-C9 Total (Damps-Konstanska et al.)	PREM-C9 Total (Hodson et al., 2019) [14]	PREM-C9 Total (Jones et al., 2020) [23]
CAT	Rho = 0.44 *p* = 0.003	Rho = 0.42 *p* = 0.03	Rho = 0.27 *p* = 0.03
HADS-Anxiety	Rho = 0.370864 *p* = 0.016	Rho = 0.30 *p* < 0.005	Rho = 0.4 *p* = 0.001
HADS-Depression	Rho = 0.387405 *p* = 0.011	Rho = 0.41 *p* < 0.005	Rho = 0.21 *p* = 0.09
